# Effect of Compound Lactic Acid Bacteria Capsules on the Small Intestinal Bacterial Overgrowth in Patients with Depression and Diabetes: A Blinded Randomized Controlled Clinical Trial

**DOI:** 10.1155/2022/6721695

**Published:** 2022-05-29

**Authors:** Fang Wei, Lei Zhou, Qingqing Wang, Guoqi Zheng, Shanshan Su

**Affiliations:** ^1^Digestive Department, Cangzhou Central Hospital, Cangzhou, China; ^2^Department of General Surgery, Cangxian Hospital, Cangzhou, China

## Abstract

**Objective:**

The objective is to explore the clinical effect of compound lactic acid bacteria capsules on the small intestinal bacterial overgrowth (SIBO) in patients with depression and diabetes.

**Methods:**

From January 2020 to January 2021, 60 SIBO patients with depression and diabetes in our hospital were selected and randomized into observation group (compound lactic acid bacteria capsules combined with escitalopram) and control group (Escitalopram) according to the odd and even numbers, 30 cases in each group. The two groups were compared in terms of SAS, SDS, levels of inflammatory factors, immune function, fasting plasma glucose (FPG), treatment effect, and the incidence of adverse reactions.

**Results:**

Both self-rating anxiety scale (SAS) and self-rating depression scale (SDS) scores in both groups showed a decline after treatment (*P* < 0.05), and the reduction was more significant in the observation group (*t* = 10.047, 17.862, all *P* ≤ 0.001). Both IL-2 and TNF-*α* in both groups showed a decline after treatment (*P* < 0.05), and the reduction was more greater in the observation group in relative to the control group (*P* < 0.05). CD3+ and CD+4 in both groups showed an increase after treatment (*P* < 0.05), and the increase was more greater in the observation group as compared to the control group (*P* < 0.05). After treatment, the FPG levels of patients in both groups showed a decline (*P* < 0.05), and the reduction of FPG levels was more significant in the observation group than that in the control group (*t* = 3.948, *P* ≤ 0.001). The control group experienced a remarkably higher incidence of adverse reactions.

**Conclusion:**

The compound lactic acid bacteria capsule is a boon for SIBO patients with depression and diabetes. It can mitigate depression symptoms, improve immune function, reduce the level of inflammatory factors, and lower the FPG levels, along with fewer adverse reactions and robust effects.

## 1. Introduction

Depression, a common mental disorder, is characterized by symptoms such as affective, cognitive, psychomotor, or autonomic disorders, which poses threats on work, study, and interpersonal relationships. The predisposing factors of depression are complex and diverse such as biochemistry, neuroendocrine and genetics, and psychosocial factors. The main manifestations are different degrees of depression and decreased interest and even self-injury and suicidal behavior in severe cases [1]. Diabetes is a common chronic disease, and depression is twice as common in individuals with diabetes as in the general population [2]. The comorbidity of depression and diabetes is associated with poor outcomes and may share biological origins [3]. In recent years, studies have found that depression is associated with small intestinal bacterial overgrowth (SIBO). Long-term depression will cause gastrointestinal reactions such as anorexia, abdominal distension, and loss of appetite and further develop into SIBO [4]. The improvement of the quality of life and relief of symptoms for SIBO patients with depression and diabetes is the priority in clinical setting. Escitalopram was previously the mainstay for the treatment of depression, which is beneficial to improve the symptoms of depression. And alternative medicine (CAM) is often used in order to alleviate these problems [5–9]. The compound lactic acid bacteria capsule is a drug for the treatment of intestinal flora disorders. This study sets to explore the effect of compound lactic acid bacteria capsules on the SIBO by enrolling 60 patients with depression and diabetes in our hospital from January 2020 to January 2021.

## 2. Materials and Methods

### 2.1. General Information

A total of 60 cases of SIBO patients with depression and diabetes admitted to our hospital from January 2020 to January 2021 were selected. Random envelopes were issued from serial 1 to 60 and placed in the envelopes. The grouping was based on the number of odd or even numbers of the cards each person held, with 30 cases each.

### 2.2. Inclusion and Exclusion Criteria

Inclusion criteria are as follows: (1) The patient was diagnosed as SIBO with depression after clinical examination and breath test [10, 11], and the diagnosis of diabetes was in accordance with the guidance of 1999 WHO criteria; (2) informed the patients and their family of the purpose and significance of the study, obtained the patient's signed informed consent form, and obtained the approval from ethics committee; (3) can communicate normally and cooperate to complete the study; and (4) the clinical data is complete. Exclusion criteria are as follows: (1) damage to vital organs; (2) patients with other types of mental illness; (3) cancer patients; (4) patients with long-term drinking history; (5) poor compliance or failure to persist until the end of the study; (6) those who had an allergic reaction to the study drug; (7) those who had immune system diseases or coagulation abnormalities; (8) pregnant women; and (9) those who had systemic infectious diseases. The study was reviewed and approved by Cangzhou Central Hospital Ethics Committee (approval no. 2019-273-12).

### 2.3. Method

The control group was given escitalopram orally (Jilin Province West Point Pharmaceutical Technology Development Co., Ltd.; National Medicine Standard H20140106; specification: 10 mg∗7 tablets), 10 mg/time, once a day. The observation group was given compound lactic acid bacteria capsule (Jiangsu Meitong Pharmaceutical Co., Ltd.; National Medicine Standard H19980184; specification: 0.33 g × 10 capsules/box) on the basis of the control group, 3 times/d at a dose of 0.66 g/time. The treatment period of both groups was 1 week. (Compound lactic acid bacteria capsules are not chemical preparations, but live bacteria preparations. Packed in film enteric-coated capsules, it is not affected by gastric acid and directly reaches the intestines for reproduction to exert its pharmacological effects. And they are not absorbed by the intestinal tract, and 1 week is therefore deemed a treatment cycle.) All participants, statistician, and investigators were blinded to the allocation of the patients and the type of medicines.

### 2.4. Observation Indicators

The anxiety and depression, inflammatory factor levels, and immune function before and after treatment, treatment effectiveness, and adverse reactions were compared. (1) The SAS scale was used to evaluate the anxiety, which includes 20 items, and scored using a 4-likert scale; the higher the score, the more severe the anxiety; the SDS scale was used to assess the depression, which includes 20 items, and scored using a 4-likert scale; the higher the score, the more severe the depression [12]. (2) 5 mL fasting peripheral venous blood in the morning was collected, centrifuged at 3000 r/min for a total of 10 minutes, and the serum was separated. Serum IL-2 and TNF-*α* were detected by ELISA, and the kit was provided by Shanghai Bohu Biotechnology Co., Ltd. (3) Immune function indexes CD3+, CD+4, CD8+, and CD4+/CD8+ were detected by flow cytometer BD FACSCalibur cell counter (Shanghai Musen Biotechnology Co., Ltd.). (4) fasting plasma glucose (FPG) was measured by a Hitachi LST008 analyzer (Hitachi High-Technologies). (5) Efficacy criteria are as follows: If the SAS and SDS reduced by≥75%, and the clinical indicators returned to normal, it is considered markedly effective. If the SAS and SDS reduced by 50%~75%, and the inflammatory index and immune function have been improved but did not return to normal, it is considered effective. If the patient's symptoms changed little before and after treatment or worsened, it is considered ineffective. The total effective rate = markedly effective rate + effective rate [13].

### 2.5. Statistical Methods

Statistical analysis was done by SPSS22.0 software package. The counting data and measurement data were expressed as *n*(%) and (x ± s), respectively, which were analyzed by X^2^ and *t* test. A *P* value of <0.05 indicates that the difference is statistically significant.

## 3. Results

### 3.1. Participants Profiles

**Figure 1:**
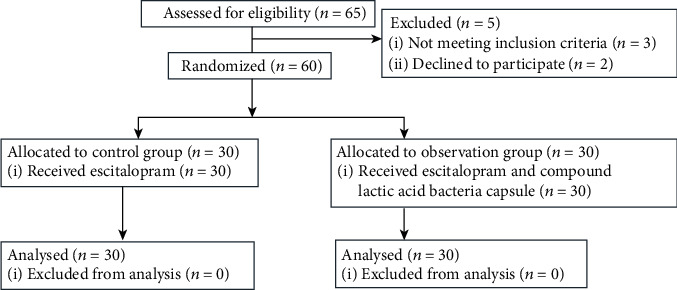
Study flowchart.

In the observation group, male: female =16 : 14, aged 23-62 (43.59 ± 4.32) years old, and the body mass index was (23.37 ± 1.31) kg/m^2^; in the control group, male: female was 17 : 13, aged 22-64 (43.31 ± 4.57) years old, and the body mass index was (23.33 ± 1.24) kg/m^2^. The baseline information of the two groups were similar (*P* > 0.05) (Figure 1).

### 3.2. Comparison of SAS and SDS Scores

Both SAS and SDS scores in both groups showed a decline after treatment (*P* < 0.05), and the reduction was greater in the observation group (*P* < 0.05) (see Table 1).

### 3.3. Comparing the Levels of Inflammatory Factors

**Figure 2:**
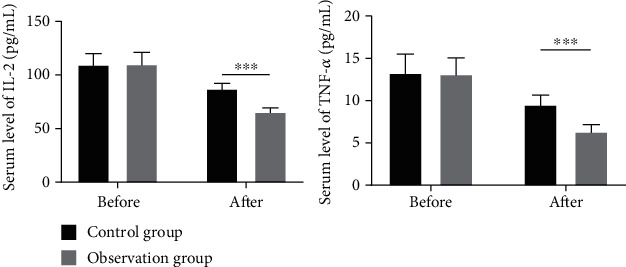
Comparison of the levels of inflammatory factors between the two groups (pg/ml, $\overset{¯}{x}\pm s$). Note: ∗∗∗represents the comparison between the two groups after treatment.

IL-2 and TNF-*α* in both groups showed a decline after treatment, whereas CD3+ and CD4+ showed an increase (*P* < 0.05), and the changes were greater in the observation group in relative to the control group (*P* < 0.05) (Figure 2).

### 3.4. Comparison of Immune Function Indexes

CD3+, CD+4, and CD4+/CD8+ in both groups showed an increase after treatment (*P* < 0.05), and the increase was greater in the observation group compared to the control group (*P* < 0.05) (see Table 2).

### 3.5. Comparison of the FPG Levels

After treatment, the FPG levels of patients in both groups show a decline (*P* < 0.05), and the reduction of FPG levels is greater in the observation group than that in the control group (*P* < 0.05), as shown in Table 3.

### 3.6. Comparison of the Efficacy

The observation group garners a notably higher efficacy than the control group, as shown in Table 4.

### 3.7. Comparison of Adverse Reactions

The control group experiences a remarkably higher incidence of adverse reactions, as shown in Table 5.

## 4. Discussion

In addition to persistent negative mood, patients with depression often experience gastrointestinal discomforts such as abdominal distension, acid reflux, and anorexia. They show no organic disease after examination and diagnosis in the department of gastroenterology and neurology. Nevertheless, the outcome remains undesirable after symptomatic treatment. In recent years, some scholars have found that there is a certain correlation between depression patients and the balance of intestinal microecology. When the intestinal microecology is out of balance, it will lead to aggravation of anxiety and depression symptoms, which counteracts on the gastrointestinal tract, causing microbiota imbalance [14], among which SIBO is the most common type. Consequently, it is particularly important to find a reliable and safe treatment for SIBO combined with depression.

Escitalopram is a common drug for clinical treatment of depression. As a 5-HT reuptake inhibitor, it has a certain selectivity and can effectively inhibit the presynaptic membrane of the central nervous system, which also can suppress 5-HT transporter. It is mainly used clinically for the treatment of depression, anxiety, somatization disorders, and psychotic anxiety. Despite the fewer side effects of the drug, the overall tolerance is favorable. Yet, the drug remains unsatisfactory for SIBO combined with depression. This study combined with compound lactic acid bacteria capsules on this basis and achieved excellent results. As a compound preparation, the compound lactic acid bacteria capsule is an important probiotic in the human intestine. Its main ingredients are *Lactobacillus* and *Streptococcus lactis*, which act on the body to multiply in the intestinal tract, increase the production of lactic acid, and inhibit the reproduction of spoilage bacteria. Through the regulation of the flora, the fermentation of spoilage bacteria can be avoided, and the occurrence of flatulence in the intestine is prevented [15]. Additionally, the drug can also speed up the body's digestion and exert a certain antidiarrheal role. The combination of the two drugs plays a role in antidepression and regulation of intestinal flora and mitigates patients' related symptoms. Remarkably, the present study showed that the observation group was superior to the control group in terms of SAS and SDS and the inflammatory factor, reflecting the robust effect of the treatment program on the patients' depressive symptoms and inflammatory response. Studies have found that the main function of T lymphocytes is immunosuppression; the long-term depression will inhibit immune function to a certain extent and increase the susceptibility of mental illness and inflammation, further undermining the neuroendocrine system as the disease progresses [16]. Notably, the immune function indicators of the patients in this study improved after treatment, and the observation group witnessed more prominent progress than the control group, suggesting the value of compound lactic acid bacteria in improving the immune function of patients. Animal studies revealed that diabetic mice treated with *Lactobacillus* showed significantly decreased FPG levels [17]. In consistent with the above study, we found that patients in the observation group had significant lower FPG levels than those in the control group. In addition, *Lactobacillus* treatment was demonstrated to regulate the expression genes that are related to glucose and lipid metabolism [18]. In terms of the effectiveness and safety of the treatment in this study, the observation group outperformed the control group, suggesting the feasibility of the treatment plan. However, on one hand, due to the limitation of research time and existing conditions, and the small sample size, the large-scale research is needed; on the other hand, the observation indicators are relatively fewer; the follow-up and various outcome measures are necessary in the future to provide more clinic references. The small sample size should be stated as a major limitation of this study. Yet, this study was a pilot clinical study, and we planned to investigate our hypothesis in a minimum sample size. Moreover, the exact cause of the adverse reactions was not investigated. Hence, it is suggested that future trials should be planned with larger sample size.

## 5. Conclusion

To conclude, the compound lactic acid bacteria capsule is a boon for SIBO patients with depression and diabetes. It can mitigate depression symptoms, improve immune function, and reduce the level of inflammatory factors and FPG, with fewer adverse reactions and robust effects.

## Figures and Tables

**Table 1:** Comparison of SAS and SDS scores between the two groups (point, $\overset{¯}{x}\pm s$).

Groups	n	SAS		*t*	*P*	SDS		*t*	*P*
		Before treatment	After treatment			Before treatment	After treatment		
Observation group	30	63.14 ± 4.73	36.12 ± 2.94	26.593	≤0.001	64.26 ± 4.64	39.75 ± 2.15	26.271	≤0.001
Control group	30	63.24 ± 4.59	43.16 ± 2.46	21.097	≤0.001	64.37 ± 4.58	50.14 ± 2.36	15.122	≤0.001
*t*	/	0.083	10.047			0.101	17.862		
*p*	/	0.934	≤0.001			0.920	≤0.001		

**Table 2:** Comparison of immune function indexes between the two groups ($\overset{¯}{x}\pm s$).

Groups	*n*	CD_3_^+^ (%)	CD^+^_4_ (%)	CD_8_^+^ (%)	CD_4_^+^/CD_8_^+^
Observation group (*n* = 30)	Before treatment	57.94 ± 6.22	29.32 ± 3.41	26.69 ± 2.53	1.05 ± 0.15
	After treatment	68.23 ± 7.72	41.48 ± 4.03	27.20 ± 2.37	1.57 ± 0.26
	*t*	5.685	12.627	0.805	9.386
	*P*	≤0.001	≤0.001	0.424	≤0.001
Control group (*n* = 30)	Before treatment	57.03 ± 6.95	29.50 ± 3.63	26.32 ± 2.49	1.06 ± 0.16
	After treatment	62.49 ± 5.56	35.28 ± 3.61	26.45 ± 2.20	1.34 ± 0.10
	*t*	3.357	6.1921	0.214	8.707
	*p*	0.001	≤0.001	0.831	≤0.001
	*t*1	0.534	0.209	0.570	0.255
	*P*	0.595	0.836	0.571	0.800
	*t*2	3.305	6.284	1.270	4.296
	*P*	0.002	≤0.001	0.209	≤0.001

Note: t1 represents the comparison between the two groups before treatment, and t2 represents the comparison between the two groups after treatment.

**Table 3:** Comparison of fasting plasma glucose (FPG) between the two groups (mmol/L, $\overset{¯}{x}\pm s$).

Groups	*n*	Before treatment	After treatment	*t*	*P*
Observation group	30	10.3 ± 1.5	8.4 ± 1.0	5.736	≤0.001
Control group	30	10.8 ± 1.8	9.8 ± 1.6	2.270	0.027
*t*	/	-1.092	-3.948		
*P*	/	0.279	≤0.001		

**Table 4:** Comparison of efficacy between the two groups (%).

Groups	*n*	Markedly effective	Effective	Ineffective	Total effectiveness (%)
Observation group	30	22 (73.3)	6 (20.0)	2 (6.7)	28 (93.3)
Control group	30	16 (53.3)	5 (16.7)	9 (30.0)	21 (70.0)
X^2^	/	/	/	/	5.455
*p*	/	/	/	/	0.020

**Table 5:** Comparison of adverse reactions between the two groups (%).

Groups	*n*	Constipate	Nausea	Dizziness	Dry mouth	Adverse reaction rate (%)
Observation group	30	1 (3.3)	0 (0.0)	1 (3.3)	0 (0.0)	2 (6.7)
Control group	30	2 (6.7)	2 (6.7)	1 (3.3)	3 (10.0)	8 (26.7)
X^2^	/	/	/	/	/	4.320
*P*	/	/	/	/	/	0.038

## Data Availability

The datasets used during the present study are available from the corresponding author upon reasonable request.
